# A Freshwater Pearl Mussel Polysaccharide Alleviates Inflammatory Pain by Targeting Peripheral Cytokines and Spinal Astroglial NLRP3/GFAP Responses

**DOI:** 10.1002/fsn3.71626

**Published:** 2026-03-13

**Authors:** Chen Hu, Weiwei Xu, Yingjian Zhu, Yushan He, Jiayuan Xu, Xiaomei Wang, Furong Niu, Guangming Chen, Hongchang Zhou

**Affiliations:** ^1^ Huzhou Key Laboratory of Precise Prevention and Control of Major Chronic Diseases, School of Medicine Huzhou University Zhejiang China; ^2^ Zhejiang Aino Biological Pharmaceutical Co., Ltd. Zhejiang China; ^3^ Traditional Chinese Medical Hospital of Huzhou Zhejiang China

**Keywords:** complete freunds adjuvant (CFA), GFAP, inflammatory pain, mussel polysaccharide (MP), NLRP3

## Abstract

Mussel polysaccharide (MP), a bioactive component derived from *Hyriopsis cumingii* and *Cristaria plicata* , exhibits promising anti‐inflammatory and analgesic effects in preclinical studies. For its unclear therapeutic mechanisms, we systematically evaluated MP's efficacy and action mechanisms in a Complete Freund's Adjuvant (CFA)‐induced rat pain model. Sixty male SD rats were divided into control, CFA, CFA + celecoxib, and CFA + MP (1.62, 0.81, 0.27 g kg^−1^) groups. Treatments began 3 days post‐CFA. Paw withdrawal threshold (PWT), thermal tail‐flick latency (TFL), paw volume (PV), and paw inflammatory scores (PIS) were measured periodically. At Day 14 after drug administration, serum IL‐6/TNF‐α were quantified using ELISA, and paw histopathological and complete blood count (CBC) were analyzed. Spinal NLRP3/GFAP expression was detected via immunohistochemistry. MP dose‐dependently improved PWT/TFL, with 0.81 and 1.62 g kg^−1^ dosage groups showing the most pronounced effects. It suppressed ipsilateral/contralateral PV, lowered serum IL‐6/TNF‐α levels, and decreased the WBC count and LYMPH%. H&E staining showed attenuated neutrophil infiltration and necrosis. Additionally, MP downregulated spinal GFAP expression and attenuated NLRP3 immunoreactivity, suggesting a potential modulation of astrocyte activation and neuroinflammatory levels. MP alleviates inflammatory pain by suppressing peripheral inflammation and modulating spinal neuroinflammation, associated with attenuated astrocyte activation and NLRP3 expression. These findings highlight MP as a promising therapeutic candidate for inflammatory pain management.

## Graphical abstract

Mussel polysaccharide (MP), a novel food resource from pearl mussels, alleviates inflammatory pain in rats in a dose‐dependent manner. It acts by dually inhibiting peripheral inflammation (reducing paw swelling and pro‐inflammatory cytokines) and spinal neuroinflammation via the NLRP3 and astrocyte pathway. These findings identify MP as a promising therapeutic candidate for inflammatory pain management.

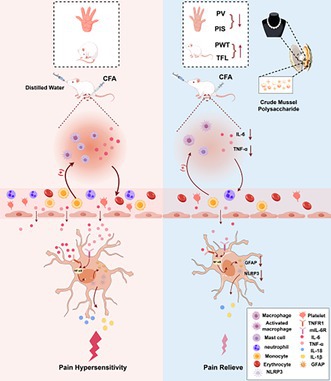

**Abbreviations**
CFA: Complete Freund's Adjuvant
GFAP: glial fibrillary acidic protein
IL‐1β: interleukin‐1β
IL‐6: interleukin‐6
LYM%: lymphocyte percentage
MCHC: mean corpuscular hemoglobin concentration
MONO%: monocyte percentage
MP: mussel polysaccharide
MP‐H: CFA + higher dosage (1.62 g kg^−1^) of mussel polysaccharide
MP‐L: CFA + lower dosage (0.27 g kg^−1^) of mussel polysaccharide
MP‐M: CFA + medium dosage (0.81 g kg^−1^) of mussel polysaccharide
MPV: mean platelet volume
NEUT%: neutrophil percentage
NLRP3: Nod‐like receptor family pyrin domain‐containing 3
PDW: platelet distribution width
PIS: Paw inflammatory scores
PV: paw volume
PWT: paw withdrawal threshold
RBC: Red blood cell
TFL: thermal tail‐flick latency
TNF‐α: tumor necrosis factor
WBC: white blood cell

## 1 Introduction

Inflammatory pain represents a significant clinical challenge arising from tissue damage‐induced neuroimmune interactions. The pathological process involves peripheral sensitization through inflammatory mediators (cytokines, prostaglandins) and central sensitization via spinal glial activation, which creates a vicious cycle of pain amplification and ultimately leads to nociceptive hypersensitivity (Ji et al. 2014). Current pharmacotherapies, particularly Nonsteroidal anti‐inflammatory drugs (NSAIDs), face limitations due to gastrointestinal complications and incomplete efficacy against neuroinflammatory components (Kuraishy et al. 2025). This therapeutic gap has spurred interest in traditional medicines with multi‐target potential and favorable safety profiles.

Among promising candidates, mussel‐derived bioactive compounds have garnered attention. The soft tissue and secretions of *Hyriopsis cumingii* and *Cristaria plicata* , which are China's endemic freshwater pearl mussels, have been pharmacologically recorded since the Ming Dynasty. Classical texts, including *Supplement to The Grand Compendium of Materia Medica* and *Materia Medica for Dietotherapy*, describe their traditional use for “yin nourishment, heat clearance, detumescence, and detoxification.” Modern biochemical analyses have identified mussel polysaccharide (MP) as the principal bioactive component, aligning with China's medicine‐food homology policy (2012) that recognizes their dual therapeutic and nutritional value. However, shellfish processing generates substantial waste (up to 80% of raw material) (Mititelu et al. 2021), which is currently disposed of through inefficient methods such as energy‐intensive calcination or repurposing as fodder meal, the latter being associated with animal digestive risks. This underutilization is particularly striking given the economic significance of these mussel species in pearl aquaculture, where residual biomass (e.g., mussel meat) retains pharmacologically active compounds but is often discarded (Suryaningtyas et al. 2025). Systematic investigation of MP's medicinal properties could not only provide cost‐effective raw materials for nutraceuticals and the development of safer analgesics but also enhance the circular economy of pearl farming by transforming waste into high‐value products.

Polysaccharide derived from freshwater mussels, particularly *Hyriopsis cumingii* and *Cristaria plicata* , have shown diverse bioactivities, including neuroprotection (Hu et al. 2010), antitumor effects (Qiu et al. 2010), and immunomodulation (Zhu et al. 2022; Qiao et al. 2015). However, their potential anti‐inflammatory and analgesic properties remain poorly characterized, despite structural similarities to marine mollusk‐derived compounds with established efficacy in anti‐inflammatory (e.g., sulfated glycosaminoglycans) (Pai et al. 2024; Krishnan et al. 2024). This investigation addresses two gaps in the existing literature on mollusk‐derived polysaccharides. Firstly, pharmacological research has predominantly focused on marine species, leaving the considerable potential of freshwater mussel polysaccharides largely untapped. Secondly, while the general anti‐inflammatory properties of some marine polysaccharides are recognized, their specific targets and efficacy within the context of neuroinflammatory pain pathways remain insufficiently elucidated (Zhu et al. 2022; Qiao et al. 2015; Pai et al. 2024; Krishnan et al. 2024). Given the rising demand for novel natural analgesics, systematic investigation into MP's multi‐target mechanisms in neuroinflammation‐related pain models is warranted.

Notably, inflammatory pain pathogenesis involves complex interactions between peripheral immune responses and central nervous system sensitization processes that may be simultaneously targeted by such bioactive compounds (Pai et al. 2024; Messina et al. 2025). Growing evidence implicates the Nod‐like receptor family pyrin domain‐containing 3 (NLRP3) inflammasome as a critical mediator of chronic inflammatory pain, where its activation can drive neuroinflammation through interleukin‐1β (IL‐1β) maturation and contribute to nociceptive signaling via astrocyte‐neuron crosstalk (Sun et al. 2024). Additionally, pro‐inflammatory cytokines, such as IL‐6 and TNF‐α are elevated in peripheral blood during inflammation, exacerbating pain hypersensitivity by sensitizing sensory neurons and promoting leukocyte infiltration (Alazragi and Baeissa 2023). Peripheral blood cell counts (e.g., neutrophils, monocytes) further reflect systemic inflammatory states, correlating with pain severity and central sensitization (Parisien et al. 2022). Given the limitations of current analgesics, such as adverse effects and incomplete efficacy, novel therapeutic strategies targeting both peripheral cytokine cascades and central pain pathways are urgently needed. MP may offer a viable solution due to its multimodal anti‐inflammatory effects. We hypothesize that MP exerts its analgesic effects through a dual mechanism: (a) attenuating peripheral inflammation by suppressing pro‐inflammatory cytokines (TNF‐α, IL‐6) and edema formation, and (b) modulating spinal neuroinflammation by inhibiting astrocyte activation (as indicated by glial fibrillary acidic protein, GFAP) and potentially interacting with neuroinflammatory pathways involving molecules such as NLRP3.

To investigate these potential mechanisms, we established a Complete Freund's Adjuvant (CFA)‐induced inflammatory pain model in rats. Animals received three different doses of MP (0.27, 0.81, and 1.62 g kg^−1^) administered daily for 14 days. Our comprehensive evaluation included: (a) inflammatory responses assessed through functional impairment, paw swelling measurement, and paw histological examination; (b) pain‐related behaviors measured by mechanical allodynia and thermal hyperalgesia; (c) peripheral inflammatory markers including complete blood counts and serum cytokine levels; and (d) central neuroinflammatory alterations focusing on spinal cord GFAP and NLRP3 expression patterns.

## 2 Materials and Methods

### 2.1 Materials

Complete Freund's Adjuvant (CFA; Catalog #7023) was acquired from Chondrex Inc. (USA). Celebrex capsules (Celebrex) were sourced from Pfizer (USA). Rat interleukin‐6 (IL‐6; #PI328) and tumor necrosis factor‐α (TNF‐α; #PT518) enzyme‐linked immunosorbent assay (ELISA) kits were procured from Beyotime (China). Von Frey filament aesthesiometer was purchased from DanMicro Global (USA) and tail‐flick analgesia meter was procured from TECHMAN INSTRUMENT (SW‐200, China). Plantar edema was quantified using a plethysmometer (TECHMAN INSTRUMENT, PV‐200, China). Complete blood cell (CBC) analysis was conducted using an automated hematology analyzer (Beckman Coulter, DxH 560, USA). Digitized slide reading of pathological sections using a fully automated digital slide scanning system (KFBIO, KF‐PRO‐120‐HI, China).

### 2.2 Preparation of Extracts and Formulation

The crude MP extract was prepared by sequential ethanol precipitation and aqueous extraction. Briefly, fresh mussel meat was minced using a meat grinder and mixed with 1–2 volumes (w/v) of 95% ethanol under continuous stirring at 25°C. The ethanol concentration was then adjusted to 70%–75% (v/v) with distilled water, and the mixture was soaked for 24 h to denature proteins and remove lipids. The precipitate was collected by pressure filtration (pressure increased at ≤ 0.1 MPa per 30 min). The resulting filter residue underwent a secondary extraction by adding 0.5–3 volumes (w/v) of distilled water with agitation for 0.5–1 h at ambient temperature and natural pH (approximately 6.5–7.0, with no external adjustment), followed by a second pressure filtration under the same controlled pressure conditions. The resulting aqueous filtrate, as a crude polysaccharide extract without further purification (e.g., dialysis or column chromatography), was subjected to spray drying (inlet temperature 85°C–180°C; outlet temperature 50°C–100°C) to obtain the final product. The crude extract yield was 3.4% (w/w, dry weight basis), with a polysaccharide content of 76.6%. MP presents as an off‐white to pale yellow granular powder exhibiting a mild shellfish‐derived odor. The bacterial endotoxin content of the MP extract was determined to be < 40 EU/mg using the gel‐clot method (Chinese Pharmacopeia, General Rule 1143). All test compounds were prepared and supplied by Ainuo Pharmaceutical Co. Ltd. (Huzhou, Zhejiang, China). The extraction protocol and the supplied pilot‐scale MP extract (prepared from three batches) are therefore representative of the established industrial process for the clinical‐grade product.

The dose of 0.27 g kg^−1^ was derived from the human clinical dose (3 g/day) via body surface area conversion. To assess dose–response, 3‐fold (0.81 g/kg) and 6‐fold (1.62 g/kg) multiples were included, based on preliminary validation. For administration, MP was initially dissolved in sterile distilled water pre‐warmed to 50°C to accelerate solubilization, followed by dilution to the desired concentrations. All suspensions were administered by oral gavage within 2 h of preparation, with gentle shaking immediately before use to ensure homogeneity.

### 2.3 Animals

Eight‐week‐old male Sprague–Dawley rats (230–260 g) were obtained from Slack Laboratory Animal Co. Ltd. (Shanghai, China). The animals were maintained under standard laboratory conditions with a 12 h light/dark cycle, ambient temperature of 23°C ± 2°C, and relative humidity of 50% ± 10%. Rats were group‐housed (3–5 animals per cage) in ventilated cages with ad libitum access to standard chow and filtered water. The use of male rats was to control for the potential confounding effects of the estrous cycle in females on pain sensitivity and inflammatory responses, thereby reducing experimental variability (Jones et al. 2025). Humane endpoints are triggered by signs of severe distress, such as > 20% weight loss, vocalization, or self mutilation, needing immediate euthanasia. All experimental procedures were conducted in strict accordance with the National Institutes of Health (NIH) Guide for the Care and Use of Laboratory Animals (8th edition, 2011) and were approved by the Institutional Animal Care and Use Committee (IACUC) of Huzhou University (No. 20230313). The study also adhered to the ethical guidelines for pain research in conscious animals established by the International Association for the Study of Pain (IASP) (Zimmermann 1983). All experimental procedures followed a predefined handling protocol to ensure methodological consistency.

### 2.4 Animal Modeling and Experimental Groups

Prior to the experiment, all rats were acclimated for 5 days and subjected to qualification testing using the paw withdrawal threshold (PWT) and thermal tail‐flick latency (TFL) tests. Animals with unqualified baseline pain thresholds were excluded.

**FIGURE 1:**
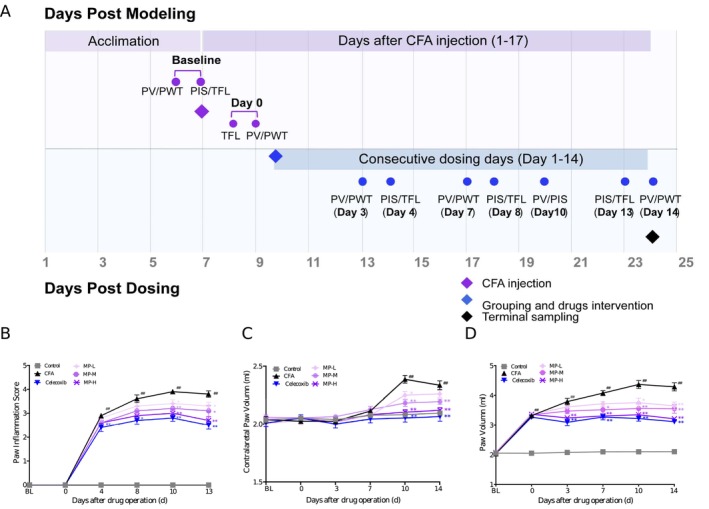
Experimental timeline and the ameliorative effects of MP on CFA‐induced plantar edema. (A) Experimental timeline with dual time axes. Baseline (BL) measurements of PWT, TFL, and PV were performed before any procedure. The upper purple axis tracks days post‐CFA modeling, with model establishment designated as Day 0. The lower blue axis tracks days post‐dosing, indicating the initiation and duration of drug treatment. Arrows mark the time points of all assessments. Key events are marked with distinct symbols: A diamond (♦) for CFA injection, a pill for drug treatment start and terminal sampling, and circles (•) for assessment time points. CFA injection induced persistent and worsening pathological changes, including paw edema, erythema, and functional impairment. (B, C) MP treatment significantly attenuated CFA‐induced local inflammation. (D) Additionally, MP ameliorated secondary contralateral paw swelling observed on Day 10. Data are presented as mean ± SEM (*n* = 10 animals per group). ^##^ *p* < 0.01, ^###^ *p* < 0.001, for CFA versus saline control; *p < 0.05, **p < 0.01 versus CFA group.

Inflammatory pain was induced under temporary anesthesia with 3% isoflurane. A single subcutaneous injection of 50 μL of Complete Freund's Adjuvant (CFA; Chondrex, catalog#7023, used as supplied at the stock concentration of 5 mg mL^−1^) was administered into the central plantar surface of the left hind paw using a 27‐gauge needle. Control rats received an equivalent injection of 50 μL of sterile saline at the same site. The experimental timeline, detailing the injection, any treatments, and all behavioral assessments, is schematically illustrated in Figure 1A.

From an initial pool of 60 qualified rats, 10 were assigned to the saline control group, while the remaining 50 received CFA. Post‐CFA baseline measurements of PWT, TFL, and PV were recorded on Days 1 and 2 post‐injection. The rats were then stratified into six treatment groups (*n* = 10 per group) based on their pain thresholds: Control (saline injection), CFA (CFA injection only), celecoxib (CFA + 0.018 g kg^−1^ celecoxib), MP‐L (CFA + 0.27 g kg^−1^ MP), MP‐M (CFA + 0.81 g kg^−1^ MP), MP‐H (CFA + 1.62 g kg^−1^ MP).

The dose of celecoxib as a positive control was based on the human equivalent dose (200 mg/day) normalized by body surface area, and aligns with efficacious doses in preclinical models (Zhang et al. 2022). Drug treatments were administered orally once daily for 14 consecutive days, on 3 days post‐CFA injection (designated as Day 1 of treatment). All drugs were suspended in pure water and administered at a fixed volume of 5 mL kg^−1^ of body weight. The control and CFA groups received equivalent volumes of pure water treatment.

Since no animals met the humane endpoint criteria or were otherwise excluded after randomization, and no major protocol deviations occurred, all randomized animals (*n* = 60) were included in the final analysis. Therefore, the results are reported based on the full intention‐to‐treat (ITT) population; a per‐protocol analysis was not conducted.

### 2.5 Paw Swelling Measurement and Inflammation Assessment

Bilateral hind paws volume (mL) was measured using a plethysmometer. At each timepoint (baseline on Day 2 post‐CFA, and Days 3, 7, 10, and 14 post‐drug administration), the measurement was performed in triplicate, and the mean value was used for analysis. Concurrently, clinical signs of CFA‐induced inflammation (edema, erythema, and localized inflammation) were systematically evaluated on the adjacent time point. Paw Inflammation Score (PIS) was employed to assess paw inflammation severity (0–4 points) according to the following criteria: 0, no edema/erythema; 1, mild erythema and slight edema; 2, moderate edema; 3, severe edema; 4, extreme edema with joint dysfunction (Zhu et al. 2018). To ensure objectivity, the clinical assessments were performed independently by two investigators who were blinded to the treatment allocations, and their scores were averaged. The experimental timeline is presented in Figure 1A.

### 2.6 Assessment of Pain‐Related Behaviors

Pain‐related behaviors were evaluated by assessing mechanical allodynia PWT and TFL.

For PWT measurement, rats were placed individually in transparent acrylic chambers (20 × 20 × 10 cm) on an elevated metal grid floor (5 × 5 mm pores). After 30 min of acclimation, mechanical stimuli were applied perpendicularly to the mid‐plantar hind paw using calibrated von Frey filaments. The 50% withdrawal threshold (g) was determined via Dixon's up‐down method (Chaplan et al. 1994; Pitcher et al. 1999). Withdrawal responses were recorded over five consecutive trials with a minimum interval of 1 min between trials. PWT was assessed at baseline (pre‐CFA injection) and pre‐MP treatment (Day 0) and at 4, 8, 13 days after drug administration.

For TFL measurement, rats were restrained in specialized holders with the dorsal tail surface exposed. A point located 3 cm proximal to the tip of the tail was marked as the site for thermal stimulation (Kříž et al. 2006). The latency (s) to a rapid tail flick response upon application of a focused radiant heat beam was recorded. TFL measurements were performed at baseline and on post‐injection Days 3, 7, and 14 after drug administration (Figure 1A). Animals exhibiting baseline TFL values outside the range of 3–10 s were excluded from the experiment to ensure consistent baseline sensitivity.

All behavioral tests were performed with the experimenter blinded to the group allocation. In addition, the order of testing for animals from different groups was randomized daily to avoid temporal bias. PWT and TFL tests were performed three replicates per rat at each time point, and the mean value was used for statistical analysis.

### 2.7 Tissue Collection and Hematology Analyses

Blood collection was performed at a single time point: 1 h after the final drug administration on Day 14 (corresponding to Day 17 post‐CFA injection). At this time, animals were deeply anesthetized via intraperitoneal injection of sodium pentobarbital (3%, 0.1 mL per 100 g). Anticoagulated whole blood samples were obtained through common carotid artery cannulation for a complete blood count (analyzed using a Beckman automated hematology analyzer) and for serum collection. Serum concentrations of IL‐6 and TNF‐α were subsequently quantified using commercially available ELISA kits, following the manufacturers' instructions.

One hour following the final drug administration on Day 14 (corresponding to Day 17 post‐CFA injection), the animals were deeply anesthetized via intraperitoneal injection of sodium pentobarbital (3%, 0.1 mL per 100 g). Anticoagulated whole blood samples were obtained through common carotid artery cannulation. A complete blood count was conducted using a Beckman automated hematology analyzer. Serum concentrations of IL‐6 and TNF‐α were quantified using commercially available ELISA kits, following the manufacturers' instructions. Samples were coded prior to assay, and the technician performing the ELISA was blinded to the treatment groups throughout the procedure.

### 2.8 Immunohistochemistry

Following blood collection, transcardial perfusion was performed with PBS followed by 4% paraformaldehyde (PFA). The lumbar spinal cord (L4–L6 segments) was dissected, post‐fixed in 4% PFA overnight and cryoprotected in 30% sucrose. Tissues were embedded in OCT compound and cryosectioned at 30 μm (Leica CM1950, −20°C). For immunohistochemistry, sections were blocked with 5% normal donkey serum and 0.3% Triton X‐100 in PBS for 1 h at room temperature, then incubated overnight at 4°C with primary antibodies: rabbit anti‐GFAP (1:100; Abcam #ab7260, USA) and rabbit anti‐NLRP3 (1:100; Abcam #ab263899, USA). Negative control sections were incubated with PBS or normal rabbit IgG instead of the primary antibodies. After PBS washes, sections were incubated with HRP‐conjugated goat anti‐rabbit IgG secondary antibodies for 2 h at room temperature (light‐protected). DAB staining was performed, followed by hematoxylin counterstaining. Sections were dehydrated, cleared in xylene, and mounted for imaging. A blinded, semi‐quantitative scoring system was employed to assess GFAP and NLRP3 immunoreactivity. Observers, unaware of sample identities, scored the sections, and a final score was obtained per subject for statistical analysis.

### 2.9 Histopathology

The left hind paw was dissected and fixed in 4% PFA for 24 h and decalcified in 5% nitric acid solution for 21 days (with the solution replaced every 3 days). After dehydration and paraffin embedding, sections were prepared and subjected to hematoxylin–eosin (H&E) staining. All section samples were scanned by a panoramic digital scanner (KFBIO, KF‐PRO‐120‐HI, China). Analysis of the images was performed using corresponding digital imaging software. A semi‐quantitative scoring system (0–3 points per criterion) was used to generate a total histopathology score for each section. All assessments were performed by two pathologists in a blinded manner. The scores from the two pathologists were averaged to yield a single total histopathology score per animal for statistical analysis.

### 2.10 Statistics

All statistical analyses were performed using SPSS 21.0 software. Continuous data are presented as mean ± standard error of the mean (SEM). Data from repeated measures over time (PWT, TFL, PV, PIS) were analyzed by two‐way repeated‐measures ANOVA, with Treatment and Time as factors. The Treatment × Time interaction was explicitly tested. Where significant effects were found, post hoc comparisons at individual time points were conducted with adjustment for multiple comparisons.

For single endpoint comparisons, parametric data were analyzed by Student's *t*‐test (two groups) or one‐way ANOVA with Dunnett's test (multiple groups). Non‐parametric or ordinal data were analyzed by the Mann–Whitney *U* test (two groups) or the Kruskal–Wallis test with Dunn's test (multiple groups). Effect sizes are reported as partial eta‐squared (ηp^2^) for ANOVA and Cohen's d for *t*‐tests. A *p*‐value < 0.05 was considered statistically significant.

## 3 Results

### 3.1 MP Treatment Alleviates PIS

Consistent with the protocol, drug administration commenced treatment Day 1, which corresponded to 3 days after CFA injection, and all animals tolerated treatment well, and no mortality or other adverse events were recorded during the study period. As shown in Figure 1B, the intraplantar CFA injection induced progressively intensifying inflammatory responses, characterized by visible erythema and edema of varying severity. Analysis of the PIS using two‐way repeated‐measures ANOVA revealed significant main effects of Treatment (*F* (5, 54) = 72.53, *p* < 0.0001) and Time (*F* (4, 216) = 297.24, *p* < 0.0001), and a significant Treatment × Time interaction (*F* (20, 216) = 16.18, *p* < 0.0001). These manifestations peaked on Day 13 and persisted through Day 16 post‐CFA injection, as evidenced by significantly elevated PIS (3.90 ± 0.10 vs. 0.00 ± 0.00 in sham controls; *p* < 0.001). Post hoc analysis showed that celecoxib exhibited early therapeutic effects, demonstrating significant reduction in inflammation by Day 4 of treatment (PIS: 2.40 ± 0.13 vs. CFA controls 2.90 ± 0.10; *p* = 0.014, Cohen's *d* = 1.17). MP‐H with statistically significant PIS suppression beginning on Day 8 of treatment (2.90 ± 0.18 vs. CFA controls 3.60 ± 0.16; *p* < 0.001), while MP‐M and MP‐L displayed delayed efficacy, achieving significant but differential anti‐inflammatory effects by Day 10 (17.9% and 12.8% PIS reduction, respectively; vs. CFA controls: *p* = 0.030 and *p* = 0.006, respectively).

### 3.2 MP Treatment Alleviates Ipsilateral and Contralateral PV

Intraplantar CFA injection induced rapid and progressive ipsilateral paw edema, peaking at Day 14 (4.37 ± 0.13 mL vs. saline controls 2.10 ± 0.01 mL; *p* < 0.001, Cohen's *d* = 7.54). Meanwhile, secondary swelling of the contralateral paw was observed at Day 14 (2.39 ± 0.03 mL vs. saline controls 2.08 ± 0.01 mL; *p* < 0.001, Cohen's *d* = 3.89) and persisted through Day 17 post CFA‐injection (Figure 1C,D). Statistical analysis using two‐way repeated‐measures ANOVA of PVs revealed highly significant Treatment × Time interactions for both the ipsilateral (*F* (25, 270) = 22.73, *p* < 0.0001) and contralateral (*F* (25, 270) = 2.41, *p* < 0.0001) paws. Significant main effects of Treatment and Time were also confirmed (ipsilateral—Treatment: *F* (5, 54) = 99.87, Time: *F* (5, 270) = 190.62; contralateral—Treatment: *F* (5, 54) = 13.69, Time: *F* (5, 270) = 26.21; all *p* < 0.0001).

Post hoc analysis showed that celecoxib significantly reduced paw swelling on Day 3, and this effect was consistently observed on Days 7, 10, and 14 of administration. Meanwhile, it also alleviated swelling in the contralateral paw on Days 10 and 14 of treatment (*p* < 0.001). MP treatment demonstrated dose‐dependent efficacy: MP‐H exhibited early anti‐edematous effects, showing significant reduction by 3 days of administration (3.24 ± 0.04 mL, vs. 3.81 ± 0.15 mL in CFA group, *p* = 0.002). MP‐M and MP‐L groups displayed progressive therapeutic effects, achieving significant edema reduction at Days 10 and 14, respectively (*p* < 0.01). Notably, both MP‐M and MP‐H treatments fully suppressed secondary contralateral inflammation from Day 10 through 14, whereas MP‐L treatment significantly attenuated contralateral paw swelling specifically on Day 10 (2.25 ± 0.03 mL vs. 2.39 ± 0.03 mL in CFA group, *p* = 0.009, Cohen's *d* = 1.32), but showed no significant effects at other time points (*p* > 0.05, Figure 1D).

### 3.3 MP Treatment Ameliorated Pain‐Like Behaviors

Compared to saline‐injected controls, CFA‐injected rats exhibited progressive thermal hyperalgesia and mechanical allodynia, as evidenced by the sustained reductions in the PWT and TFL from 24 h through Day 16/17 post‐CFA injection. Analysis of PWT using two‐way repeated‐measures ANOVA revealed significant main effects of Treatment (*F* (5, 54) = 25.01, *p* < 0.0001) and Time (*F* (4, 216) = 60.21, *p* < 0.0001), and a significant Treatment × Time interaction (*F* (20, 216) = 3.79, *p* < 0.0001). Analysis of thermal hyperalgesia (TFL) also showed significant main effects of Treatment (*F* (5, 54) = 29.14, *p* < 0.0001) and Time (*F* (4, 216) = 96.88, *p* < 0.0001), along with a significant Treatment × Time interaction (*F* (20, 216) = 11.00, *p* < 0.0001). The PWT decreased from 26.74 ± 1.52 to 4.04 ± 0.39 g, while the TFL declined from 6.42 ± 0.21 to 4.65 ± 0.16 s (all *p* < 0.001 vs. saline‐controls at corresponding time points). At the endpoint, these differences corresponded to extremely large effect sizes, with Cohen's *d* values of 5.57 for PWT and 3.73 for TFL. This confirmed successful establishment of inflammatory pain. Post hoc analysis showed that, as predicted, celecoxib treatment significantly attenuated these nociceptive responses (*p* < 0.001 vs. CFA group), thereby validating the model's reliability.

**FIGURE 2:**
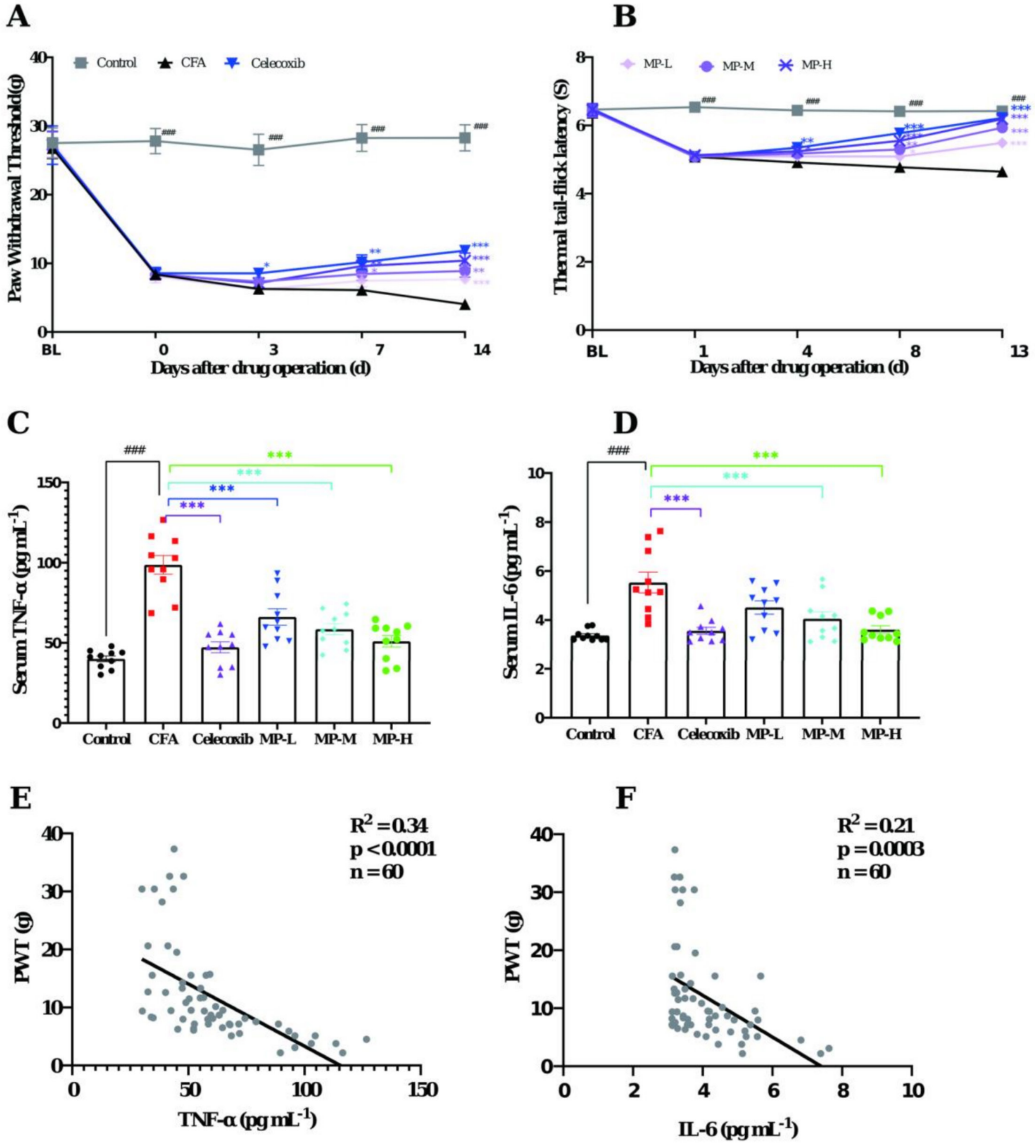
MP attenuates CFA‐induced pain hypersensitivity and normalizes serum pro‐inflammatory cytokines, which correlate with behavioral outcomes. (A) PWT assessed by von Frey filaments. (B) TFL measured by thermal tail‐flick latency test. (C, D) Serum levels of pro‐inflammatory cytokines (C) IL‐6 and (D) TNF‐α measured by ELISA. (E) Pearson correlation analysis between serum TNF‐α levels and mechanical PWT across all animals (*n* = 60). The solid line represents the linear regression fit (*R* ^2^ = 0.34, *p* < 0.0001). (F) Pearson correlation analysis between serum IL‐6 levels and mechanical PWT (*n* = 60). The solid line represents the linear regression fit (*R* ^2^ = 0.21, *p* = 0.0003). Data are presented as mean ± SEM. Statistical symbols are as defined in Figure 1.

During the early treatment period (Days 3–4), the MP groups showed mild improvements in PWT and TFL, but without statistical significance compared to CFA (all *p* > 0.05). By Days 7–8 of treatment, both MP‐H and MP‐M groups effectively prevented CFA‐induced hypersensitivity, demonstrating progressive recovery of mechanical sensitivity (Day 7, PWT: 9.60 ± 0.87 g and 8.44 ± 0.92 g) and thermal thresholds (Day 8, TFL: 5.55 ± 0.08 s and 5.30 ± 0.10 s), with sustained efficacy through Days 13–14 (all *p* < 0.001 vs. CFA controls, Cohen's *d* = 2.42 and 2.97). The MP‐L group exhibited delayed mechanical analgesia, reaching significance only on Day 14 (PWT: 7.67 ± 0.36 g, *p* < 0.001, Cohen's *d* = 3.03), but maintained consistent thermal analgesia at both evaluation timepoints (Day 8, 5.09 ± 0.10 s, *p* < 0.01, Cohen's *d* = 1.07; Day 13, 5.49 ± 0.13 s, *p* < 0.001, Cohen's *d* = 1.87) (Figure 2A,B).

### 3.4 MP Ameliorated Hematological Parameters Abnormalities

Complete blood count analysis revealed significant hematological disturbances in the CFA‐treated group compared to saline‐treated controls (Table 1). Specifically, CFA induced marked leukocytosis, characterized by elevated white blood cell counts (19.78 ± 1.00 × 10^9^ L^−1^), increased neutrophil percentage (46.68% ± 3.37%) and heightened platelet counts (1347.90 ± 38.1 × 10^9^ L^−1^) (all *p* < 0.001), alongside a significant reduction in lymphocyte percentage. Celecoxib treatment effectively normalized these alterations (*p* < 0.01 for neutrophil and lymphocyte percentages; *p* < 0.001 for WBC and platelet counts). Similarly, treatment with MP at all three doses significantly attenuated the CFA‐induced hematological changes. The high‐dose MP showed a pronounced effect, particularly in reducing WBC counts to 12.25 ± 1.27 × 10^9^ L^−1^ (*p* < 0.001). Notably, no significant differences were observed in erythrocyte‐related parameters or platelet indices among all groups.

**TABLE 1:** Hematological parameters in the groups (mean ± SEM, *n* = 10).

Parameters	Control	CFA	Celecoxib	MP‐L	MP‐M	MP‐H
Total WBC (10^9^ L^−1^)	8.73 ± 0.63	19.78 ± 1.00^###^	11.12 ± 1.23***	14.38 ± 1.46**	14.72 ± 1.35**	12.25 ± 1.27***
Differential counts (%)						
(i) Neutrophils	17.73 ± 0.79	46.68 ± 3.37^###^	32.22 ± 2.81**	31.03 ± 2.22**	35.27 ± 1.63**	32.97 ± 4.00*
(ii) Lymphocytes	75.36 ± 1.03	44.81 ± 3.46^###^	60.08 ± 2.93**	60.82 ± 2.33**	56.22 ± 1.56**	59.02 ± 4.10*
(iii) Monocytes	5.69 ± 0.35	7.39 ± 0.56^#^	6.54 ± 0.55	6.76 ± 0.39	7.53 ± 0.28	6.95 ± 0.53
(iv) Eosinophils	1.24 ± 0.13	1.07 ± 0.11	1.13 ± 0.13	1.37 ± 0.16	0.95 ± 0.13	1.04 ± 0.13
(v) Basophils	0.04 ± 0.02	0.05 ± 0.02	0.03 ± 0.02	0.02 ± 0.01	0.03 ± 0.02	0.02 ± 0.01
RBC (^1012^L^−1^)	6.96 ± 0.17	7.25 ± 0.14	7.33 ± 0.09	7.12 ± 0.14	7.26 ± 0.14	7.14 ± 0.18
Hemoglobin (g dL^−1^)	138.40 ± 2.71	140.20 ± 2.95	146.30 ± 1.33	139.30 ± 2.56	142.30 ± 2.47	139.20 ± 4.01
MCHC (g dL^−1^)	19.90 ± 0.17	19.33 ± 0.19^#^	19.97 ± 0.23	19.61 ± 0.17	19.55 ± 0.25	19.48 ± 0.22
Platelets (10^9^ L^−1^)	809.78 ± 18.82	1347.90 ± 38.1^###^	1051.50 ± 46.78***	1117.50 ± 81.28*	1109.40 ± 74.24*	1109.50 ± 75.26*
MPV (fL)	6.27 ± 0.07	6.45 ± 0.06	6.76 ± 0.10	6.32 ± 0.07	6.54 ± 0.07	6.70 ± 0.10
PDW (%)	14.85 ± 0.06	14.87 ± 0.03	14.88 ± 0.04	14.78 ± 0.04	14.82 ± 0.03	14.94 ± 0.05

*Note:* #*p* < 0.05, ###*p* < 0.001 for CFA group versus control group; *p < 0.05, **p < 0.01, ***p < 0.001 versus CFA group (one‐way ANOVA followed by Dunnett's test).

The observed leukocytosis with neutrophilia in the CFA group reflects a sustained systemic inflammatory response. MP's ability to attenuate this response, particularly the reduction in total leukocyte and neutrophil counts, reinforces its systemic anti‐inflammatory action.

### 3.5 MP Attenuated CFA‐Induced Upregulation of Serum TNF‐α and IL‐6

As shown in Figure 2C,D, a profound elevation in serum pro‐inflammatory cytokines was observed 17 days after CFA injection compared to saline‐controls. Specifically, TNF‐α levels were robustly elevated to 98.61 ± 5.86 pg mL^−1^ (Cohen's *d* = 4.27) from a control level of 40.04 ± 1.82 pg mL^−1^, while IL‐6 levels increased to 5.53 ± 0.42 pg mL^−1^ (Cohen's *d* = 2.26) from 3.37 ± 0.07 pg mL^−1^ (both *p* < 0.0001). Celecoxib treatment showed significant inhibitory effects on both TNF‐α (47.22 ± 3.39, *p* < 0.0001 vs. CFA group; Cohen's *d* = −3.39) and IL‐6 production (3.56 ± 0.15, *p* = 0.0003 vs. CFA group; Cohen's *d* = −1.98).

Fourteen‐day administration of MP demonstrated dose‐dependent anti‐inflammatory effects. The high (1.62 g kg^−1^) and medium (0.81 g kg^−1^) doses significantly reduced both cytokines (TNF‐α: 51.11 ± 3.64 pg mL^−1^, *p* < 0.0001, Cohen's *d* = 3.08 and 58.62 ± 3.47 pg mL^−1^, respectively; IL‐6: 3.60 ± 0.16, *p* = 0.0005, Cohen's *d* = 1.91 and 4.05 ± 0.28 pg mL^−1^, *p* = 0.009, Cohen's *d* = 1.30). While the low dose (0.27 g kg^−1^) effectively decreased the TNF‐α level (66.13 ± 15.93 pg mL^−1^, *p* = 0.0005, Cohen's *d* = 1.88), its effect on IL‐6 reduction (4.51 ± 0.27, *p* = 0.058, Cohen's *d* = 0.91) showed a strong trend but did not reach statistical significance.

To further investigate the relationship between systemic inflammation and pain behavior, a Pearson correlation analysis was performed using data from all experimental animals (*n* = 60). As shown in Figure 2E, a strong and significant negative correlation was observed between the serum level of TNF‐α and the PWT (*R* ^2^ = 0.3386, *p* < 0.0001). Similarly, a significant negative correlation was found between IL‐6 and the PWT (*R* ^2^ = 0.2078, *p* = 0.0003; Figure 2F). These results robustly demonstrate that the elevation of pro‐inflammatory cytokines is closely associated with the severity of mechanical hypersensitivity in this model of inflammatory pain.

### 3.6 MP Ameliorates CFA‐Induced Paw Pathological Inflammatory Damage

**FIGURE 3:**
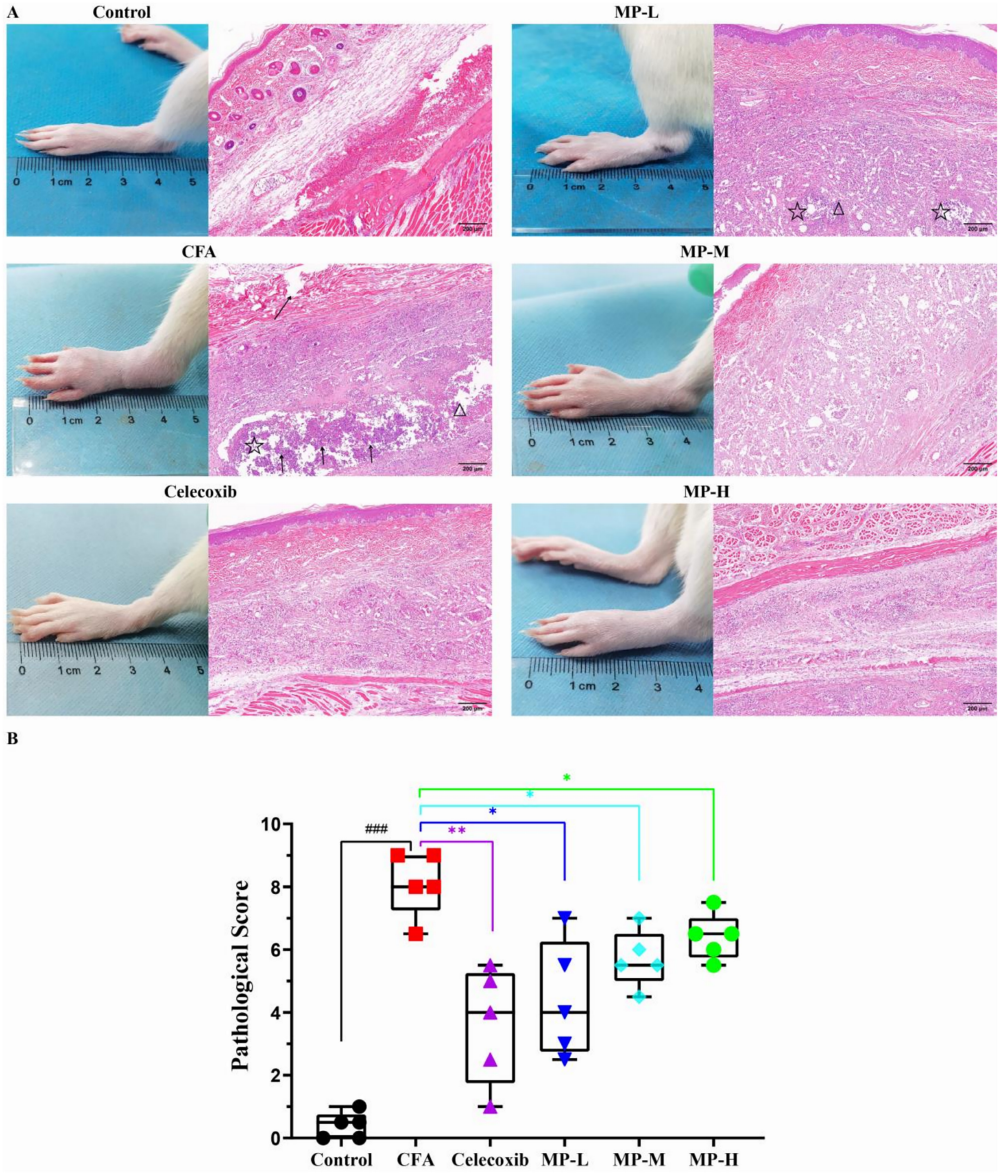
MP attenuates CFA‐induced pathological changes in paw tissues (H&E staining). (A) Representative photographs show paw edema morphology and corresponding H&E‐stained sections (10× magnification) that demonstrate the histopathological features of inflammation and tissue damage across experimental groups. As shown in the results, the control group exhibited intact skin layers (epidermis, dermis, and subcutaneous tissue) and skeletal muscle with clear cross‐striations in muscle fibers. The CFA group showed focal abscess formation (☆) with massive neutrophil infiltration (↑) and marked tissue necrosis (evidenced by pyknosis, karyolysis, and eosinophilic cytoplasm), accompanied by necrotic areas (△); diffuse inflammatory cell infiltration was observed in subcutaneous tissue and intermuscular spaces, along with muscle fiber degeneration and disruption (↗). Compared with the CFA group, celecoxib treatment significantly alleviated inflammatory lesions without obvious abscess formation or tissue necrosis, and muscle fibers were more regularly arranged with rare degeneration or rupture. In the MP‐treated groups, MP‐L (low dose, 0.27 g kg^−1^) demonstrated mild improvement with smaller abscess foci (☆), reduced neutrophil infiltration, and diminished necrotic areas (△); MP‐M (medium dose, 0.81 g kg^−1^) showed moderate improvement without obvious abscess formation but with persistent inflammatory cell infiltration and minimal necrosis; MP‐H (high dose, 1.62 g kg^−1^) exhibited significant attenuation of inflammation with markedly reduced inflammatory cell infiltration, few neutrophils, and nearly absent necrotic areas, indicating effective control of the inflammatory process. Scale bar = 200 μm. (B) Composite histopathology score per animal. The scoring system for each parameter was as follows: Edema, inflammatory cell infiltration, and degeneration & necrosis were each graded from 0 to 3 (0, absent; 1, mild; 2, moderate; 3, severe). The scores for the three parameters were summed, resulting in a total score ranging from 0 to 9. Data are presented as mean ± SEM (*n* = 5). Statistical comparisons between the high‐, medium‐, low‐dose groups and the model group were performed using the Mann–Whitney *U* test (*p < 0.05, **p < 0.01, ***p < 0.001).

H&E staining revealed distinct pathological alterations in the tissues. As shown in Figure 3A, the CFA model group exhibited severe inflammatory damage, characterized by extensive abscess formation, widespread tissue necrosis, dense neutrophil infiltration, and substantial inflammatory exudate accumulation. In contrast, the saline‐injected control group maintained normal tissue architecture without significant pathological changes (Control vs. CFA: 0.40 ± 0.19 vs. 8.10 ± 0.46, *p* < 0.0001, Cohen's *d* = 9.84).

To quantitatively substantiate these observations, a semi‐quantitative histopathological scoring system (0–3 points for each criterion: inflammatory cell infiltration, tissue necrosis, and edema) was employed (Figure 3B). The CFA model group yielded a significantly high cumulative score of 8.10 ± 0.46. Following the 14‐day treatment, all MP‐treated groups showed a marked reversal of these histopathological scores. The scores were significantly reduced to 4.40 ± 0.83, 5.70 ± 0.41, and 6.40 ± 0.33 for the MP‐H, MP‐M, and MP‐L groups, respectively (MP‐H vs. CFA: *p* = 0.0159; MP‐M vs. CFA: *p* = 0.0159; MP‐L vs. CFA: *p* = 0.0317). The lower scores in all treatment groups quantitatively reflected the observed mitigation of pathological features, such as reduced abscess size and inflammatory cell infiltration.

### 3.7 MP Downregulated GFAP Activation and NLRP3 Expression

**FIGURE 4:**
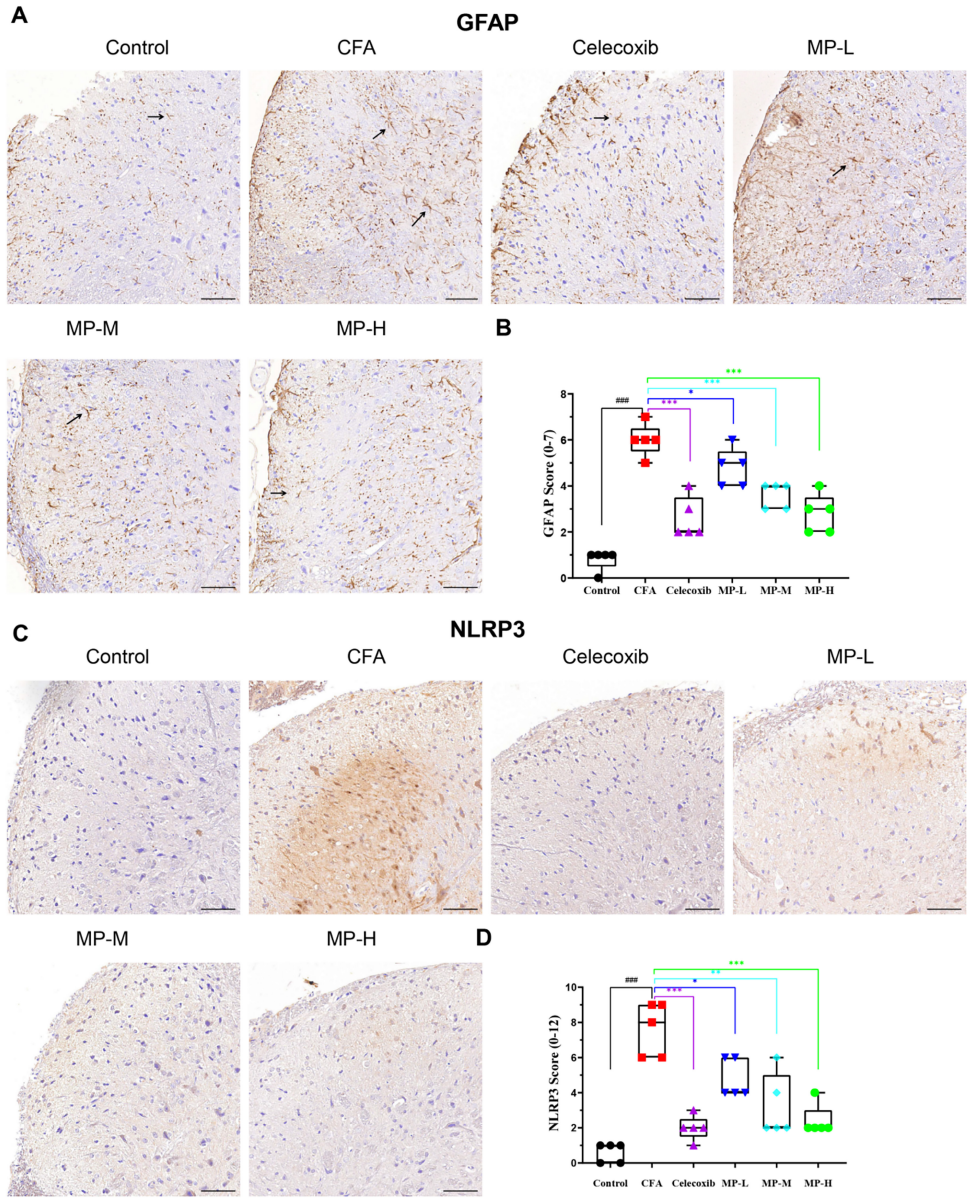
MP attenuates GFAP activation and NLRP3 expression in the spinal. (A) Representative micrographs of GFAP immunostaining. Saline‐treated controls exhibited resting astrocytes with small bodies and slender processes (→). CFA induced marked cellular hypertrophy and process thickening (↗). Celecoxib and MP treatments show a graded reduction in this reactivity compared to CFA group. (B) Quantitative analysis of GFAP immunoreactivity. Data were scored on a semi‐quantitative scale (range 0–7) and are presented as mean ± SEM (*n* = 5 per group). (C) Representative micrographs of NLRP3 immunostaining. The CFA group showed dense punctate aggregates, granular deposits, and patchy staining patterns. (D) Quantitative analysis of NLRP3 immunoreactivity. Data were scored on a semi‐quantitative scale (range 0–12) and are presented as mean ± SEM (*n* = 5 per group). Data in (B) and (D) were analyzed by one‐way ANOVA followed by Tukey's post hoc test. *p < 0.05, **p < 0.01, ***p < 0.001 versus CFA group; ###*p* < 0.001 versus control group. Scale bars = 50 μm.

Immunohistochemical analysis revealed distinct patterns of astrocyte activation and NLRP3 immunoreactivity in the spinal dorsal horn following CFA induction (Figure 4). In saline‐treated controls (Figure 4A), GFAP‐positive astrocytes remained in a quiescent state. Quantification confirmed a low baseline reactivity score. CFA injection induced robust astrogliosis, characterized by significantly enlarged cell bodies and thickened processes, which was reflected by a marked increase in the GFAP score (*p* < 0.001 vs. control). MP treatment dose‐dependently attenuated this activation (MP‐L: *p* < 0.05, MP‐M and MP‐H: *p* < 0.001, all vs. CFA group), with the MP‐H group showing a reduction comparable to that achieved by celecoxib (Figure 4B).

NLRP3 immunoreactivity was barely detectable in saline‐treated controls by immunohistochemistry (Figure 4C). CFA administration markedly enhanced NLRP3 immunostaining, appearing as dense punctate or granular deposits. This was associated with a significant rise in the NLRP3 score (*p* < 0.001 vs. control, Figure 4D). Celecoxib demonstrated potent inhibitory effects, substantially lowering the NLRP3 score (*p* < 0.001 vs. CFA group). Similarly, MP treatment produced a dose‐dependent reduction in NLRP3 immunoreactivity (MP‐L: *p* < 0.05, MP‐M: *p* < 0.01 and MP‐H: *p* < 0.001, all vs. CFA group), with the MP‐M and MP‐H groups displaying sparse punctate staining, while the MP‐L group exhibited moderate granular and patchy cytoplasmic signals (Figure 4D).

### 3.8 MP Dose‐Dependently Inhibits Peripheral and Central Inflammatory Responses

To link the systemic and central anti‐inflammatory effects of MP, we correlated its dose‐dependent reduction of serum pro‐inflammatory cytokines (TNF‐α and IL‐6) with that of spinal cord GFAP and NLRP3 expression. The rank order of treatment efficacy was identical across all measured outcomes: MP‐H≈celecoxib>MP‐M>MP‐L>CFA. Specifically, the MP‐H and MP‐M doses most significantly (*p* < 0.001) and substantially reduced serum TNF‐α and IL‐6 to near‐control levels, and also most completely suppressed both astrocytic activation and NLRP3 immunoreactivity. Conversely, the MP‐L dose, which resulted in a moderate (and for IL‐6, statistically non‐significant, *p* = 0.058) reduction in cytokines, aligned with its intermediate phenotypic effect—only partially reversing CFA‐induced astrogliosis and leaving clearly detectable NLRP3 signals. This direct, dose‐dependent correspondence demonstrates that the efficacy of MP in inhibiting peripheral cytokines is closely paralleled by its potency in suppressing key neuroinflammatory pathways in the spinal cord.

## 4 Discussion

The CFA‐induced inflammatory pain model has been widely utilized to study chronic inflammatory responses due to its well‐characterized pathological features, including persistent hyperalgesia, edema, and localized leukocyte infiltration (Butler et al. 1992). Our study demonstrates that MP administration produces significant anti‐inflammatory and analgesic effects in a time‐ and dose‐dependent manner. Higher doses of MP (1.62 and 0.81 g kg^−1^) exhibited rapid anti‐edema effects as early as Day 4 post‐treatment, while the lowest dose (0.27 g kg^−1^) required prolonged administration for comparable efficacy, suggesting early modulation of vascular permeability or inflammatory mediator release (Qiao et al. 2016; Xiang et al. 2022). Histopathological analysis confirmed MP's tissue‐protective effects, showing reduced abscess formation, necrosis, and inflammatory infiltration. These findings are consistent with the reported ability of shellfish‐derived polysaccharide derivatives to promote macrophage polarization toward the anti‐inflammatory M2 phenotype (Wu et al. 2021), suggesting a potential mechanism for MP's observed anti‐inflammatory and tissue‐reparative actions.

MP also demonstrated systemic effects, significantly attenuating edema in both ipsilateral and contralateral paws by Day 10 (Figure 1C,D). This was supported by reduced serum levels of TNF‐α and IL‐6, along with normalized hematologic parameters, with decreased NEUT%, total WBCs, and platelet counts (Figure 2C,D; Table 1). The suppression of these key cytokines correlated with rapid edema resolution and a decline in CFA‐induced thrombocytosis, indicating that MP interrupts the cytokine‐mediated cascade of vascular permeability, leukocyte recruitment, and secondary thrombocytosis. The significant reduction in total leukocyte and neutrophil counts indicates that MP treatment effectively attenuated the systemic inflammatory response associated with CFA‐induced pain. This systemic anti‐inflammatory effect is clinically relevant, as peripheral inflammation is known to contribute to both local tissue pathology and the facilitation of pain signaling pathways. Notably, significant negative correlations between serum TNF‐α/IL‐6 levels and PWT (Figure 2E,F) further strengthen the link between the attenuation of systemic inflammation and the pain behavior. Interestingly, the fact that TNF‐α explained a greater proportion of the variance in pain behavior (*R* ^2^ = 0.34) than IL‐6 (*R* ^2^ = 0.21) might suggest that TNF‐α could play a more dominant or upstream role in mediating mechanical hypersensitivity in this specific context. This is consistent with the well‐established notion that TNF‐α often sits atop the inflammatory cytokine cascade.

The compelling evidence of MP's systemic anti‐inflammatory action and its correlation with pain relief prompted us to investigate whether its analgesic effects also involve modulation beyond the periphery. Given that persistent peripheral inflammation is a known driver of central sensitization, we hypothesized that MP might disrupt this cascade by targeting spinal cord neuroinflammation. Supporting this hypothesis, we observed a clear temporal dissociation: the onset of analgesia for all MP doses (Figure 2A) lagged behind the resolution of peripheral edema (Figure 1B,C), suggesting an indirect, central mechanism.

Critically, this peripheral‐to‐central relationship was further substantiated by a consistent, dose‐dependent hierarchy: the potency of MP in reducing serum TNF‐α and IL‐6 was closely mirrored by its efficacy in suppressing spinal cord GFAP and NLRP3 immunoreactivity. The high‐ and medium‐dose MP, which most substantially reduced peripheral cytokines, also produced the strongest suppression of astrocyte activation and the reduction in spinal NLRP3 expression. Conversely, the low‐dose MP, which only modestly mitigated peripheral inflammation, produced a correspondingly intermediate reversal of these neuroinflammatory markers. This coherent, dose‐for‐dose correspondence across compartments strongly indicates that the degree of peripheral cytokine inhibition is a key determinant for the subsequent suppression of central sensitization pathways. The parallel amelioration of hematological parameters (e.g., leukocytosis, neutrophilia) further supports this integrated view, suggesting that mitigating the systemic inflammatory milieu contributes to breaking the cycle of peripheral‐central crosstalk that sustains chronic pain states.

**FIGURE 5:**
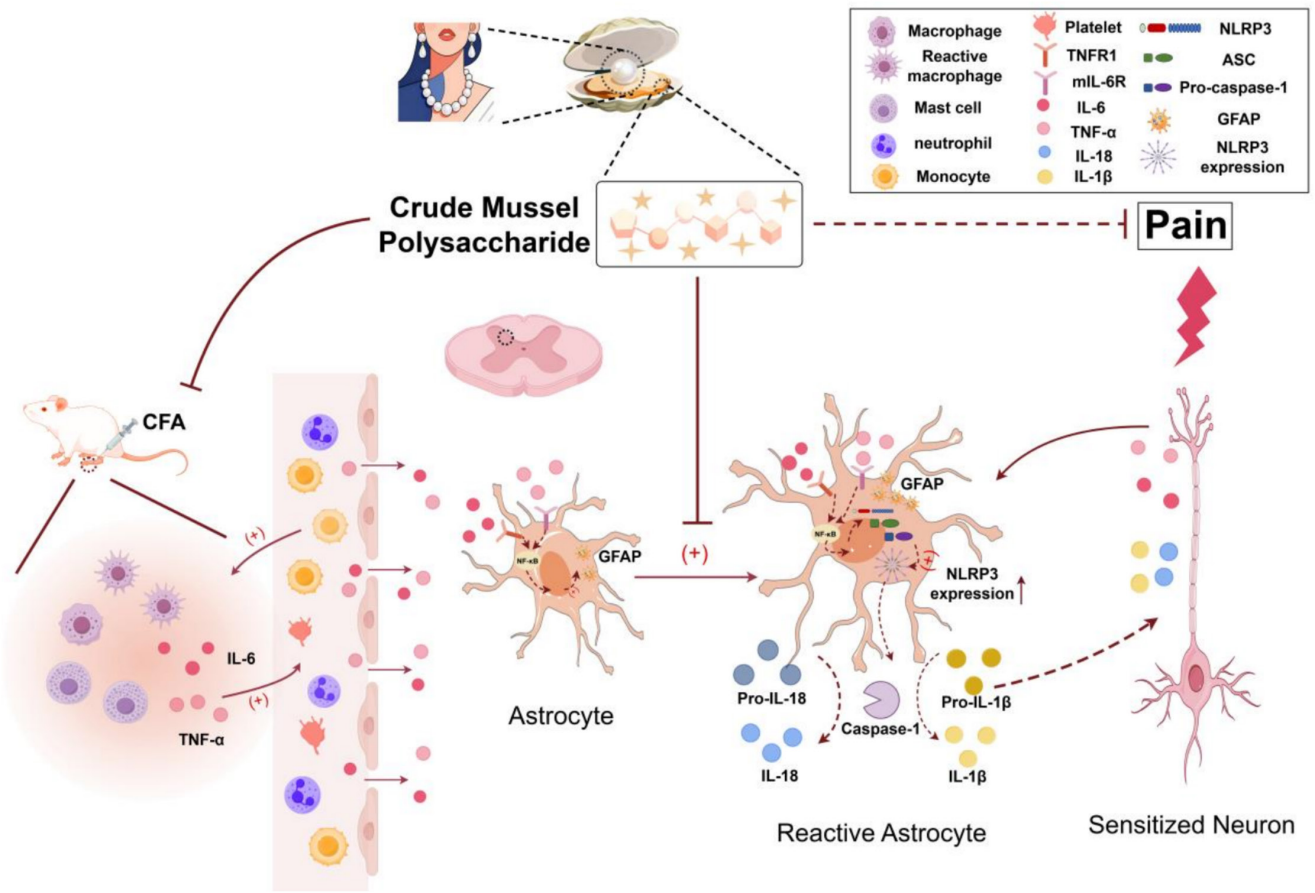
Proposed mechanism of MP‐mediated analgesia in CFA‐induced inflammatory pain at the spinal level. CFA‐induced peripheral inflammation elevates levels of pro‐inflammatory cytokines (TNF‐α, IL‐6), leukocytes, and lymphocytes. These factors cross the blood‐nerve barrier, activating spinal astrocytes (as indicated by increased GFAP) and upregulating the expression of NLRP3, a key component of the inflammasome pathway. Based on the established role of this pathway in neuroinflammation, we propose a model in which increased NLRP3 may lead to inflammasome assembly and subsequent caspase‐1‐dependent release of IL‐1β and IL‐18, contributing to neuronal sensitization and pain hypersensitivity. Our data suggest that the freshwater‐derived MP attenuates this pain signaling cascade by suppressing peripheral cytokine production and subsequent astrocyte activation and NLRP3 expression, ultimately alleviating pain behavior.

This peripheral‐to‐central signaling cascade aligns with established neuroimmune crosstalk in inflammatory pain (Aby et al. 2022). As summarized in Figure 5, MP's central effects include suppressing GFAP upregulation, a hallmark of astrocyte activation. By doing so, MP may restore glial‐neuronal communication and attenuate the release of pro‐algesic factors, enhanced neurotransmitter release, and disrupted glutamate homeostasis (Ji et al. 2013; Tang et al. 2021; Guan et al. 2025). The delayed analgesia (Days 7–14) relative to peripheral effects (Day 4) supports that MP's action progresses from the periphery to the central nervous system. MP also downregulated the expression of NLRP3, a core component of the inflammasome pathway and a potential driver of neuroinflammation. Given that NLRP3 expression is often regulated by pro‐inflammatory signals such as TNF‐α and IL‐6 during the priming phase (Cheng et al. 2023; Lin et al. 2022; Choi et al. 2010), the observed reduction in these peripheral cytokines may contribute to the decreased spinal NLRP3 expression. This modulation, alongside the suppression of GFAP, may help interrupt the self‐perpetuating neuroinflammatory loop. The dose‐dependent efficacy of MP highlights its unique pharmacological profile. Unlike classical NSAIDs (Bindu et al. 2020) that exert immediate peripheral effects, MP's gradual onset suggests a multimodal mechanism involving sequential neuroinflammatory modulation. This raises a pivotal question: whether its central action results from reduced peripheral inflammation decreasing central sensitization, or direct penetration of active components across the blood‐nerve barrier.

Despite these insights, it is important to acknowledge the limitations of our study, which also define the critical gaps for future research. First, while we observed a clear temporal sequence from peripheral anti‐inflammation to central analgesia, our experimental design cannot definitively establish a causal link between the two. Resolving this distinction—whether the central action results from reduced peripheral inflammation or from direct penetration of active components across the blood–brain barrier—is critical for therapeutic optimization. Current evidence supports the former, but future studies directly measuring MP's CNS bioavailability and specific neuronal protection markers are needed to distinguish these possibilities. Second, the precise bioactive compounds within MP responsible for the observed effects remain uncharacterized, as the extract was used as a whole mixture. Third, while our data demonstrate upregulation of NLRP3 expression in the spinal cord, we did not measure downstream effectors of inflammasome activation (such as cleaved caspase‐1 or mature IL‐1β/IL‐18). Therefore, our conclusions regarding functional inflammasome assembly and activity remain inferential and require validation in future studies. Fourth, although we propose mechanisms based on protein expression (GFAP, NLRP3), direct functional evidence linking these specific targets to the behavioral outcomes is still needed.

Addressing these limitations requires an integrated research strategy. To disentangle the central versus peripheral site of action, cell‐specific knockout models will be essential (Ohm et al. 2024). Concurrently, the active pharmacophores must be identified and their clear structure–activity relationships established through metabolomics and glycomics profiling (Jiang et al. 2022). Furthermore, the identity of the primary molecular targets (e.g., TLR4 signaling and its potential link to NLRP3 expression) warrants further investigation (Guan et al. 2025). Finally, the potential confounding effect of the inherent endotoxin background (< 40 EU/mg) in the crude extract, though deemed negligible in this oral study, should be definitively ruled out in future confirmatory studies using purified or endotoxin‐depleted preparations. Concurrently, well‐designed trials in distinct pain subtypes (neuropathic vs. inflammatory) are needed to translate these mechanistic insights into clinical efficacy.

Thus, realizing the full potential of MP as a functional food resource depends on addressing these mechanistic gaps. Despite challenges in target identification, MP possesses distinct translational advantages. Its established safety profile as a functional food ingredient facilitates regulatory approval. At the same time, the inherent multitarget effect of polysaccharide enables simultaneous modulation of multiple pain pathways, and its dual peripheral‐central actions offer comprehensive, disease‐modifying potential unmatched by single‐target analgesics. As a paradigm of naturally‐derived traditional medicine, MP represents a promising bridge between dietary intervention and pharmacological therapy for chronic inflammatory conditions. Its development exemplifies the emerging trend of harnessing natural product complexity as a therapeutic advantage rather than a limitation.

## 5 Conclusions

This study confirms that MP alleviates CFA‐induced inflammatory pain via dual mechanisms: (a) peripherally, it reduces paw swelling, improves PWT/TFL, decreases leukocyte infiltration, and lowers serum TNF‐α/IL‐6 levels; (b) centrally, it suppresses spinal GFAP activation and reduces NLRP3 expression. The effects are dose‐dependent and time‐sensitive. These findings demonstrate MP's anti‐inflammatory and analgesic properties, supporting its potential as a therapeutic agent for inflammatory pain through combined peripheral and central actions.

## Author Contributions

**Chen Hu:** conceptualization (equal), investigation (equal), methodology (equal), writing – original draft (equal). **Weiwei Xu:** conceptualization (supporting), funding acquisition (lead). **Yingjian Zhu:** data curation (equal), investigation (equal). **Yushan He:** investigation (equal), methodology (equal). **Jiayuan Xu:** data curation (equal), formal analysis (equal). **Xiaomei Wang:** investigation (equal), methodology (equal). **Furong Niu:** conceptualization (equal), supervision (equal). **Guangming Chen:** conceptualization (equal), writing – review and editing (equal). **Hongchang Zhou:** conceptualization (equal), supervision (equal), writing – original draft (equal).

## Funding

This research was investigator‐initiated but funded by Zhejiang Aino Biological Pharmaceutical Co. Ltd.

## Ethics Statement

This study was approved by the Animal Welfare and Ethics Committee of Huzhou University (No. 20230313). We confirmed that all experiments in this study were performed in accordance with the relevant guidelines and regulations. All the procedures of the study are followed by the ARRIVE guidelines.

## Conflicts of Interest

The funders provided financial support for this study and participated in reviewing the drug preparation methodology, but had no role in study design, data collection, analysis, or interpretation of the results. The authors retained full control of all scientific decisions. W.X., an employee of Zhejiang Aino Biological Pharmaceutical Co. Ltd., played a role in funding this research and is listed as the second author in the manuscript. All the other authors declare no competing interests.

## Data Availability

The data that support the findings of this study are openly available in Figshare at https://doi.org/10.6084/m9.figshare.30575396.
